# Conservation laws and exact solutions of the $(3+1)$-dimensional Jimbo–Miwa equation

**DOI:** 10.1186/s13662-021-03578-4

**Published:** 2021-09-23

**Authors:** Jalil Manafian, Elnaz Alimirzaluo, Mehdi Nadjafikhah

**Affiliations:** 1 Department of Applied Mathematics, Faculty of Mathematical Sciences, University of Tabriz, Tabriz, Iran; 2 Natural Sciences Faculty, Lankaran State University, 50, H. Aslanov str., Lankaran, Azerbaijan; 3 School of Mathematics, Iran University of Science and Technology, 16844 Tehran, Iran

**Keywords:** 17B45, 20G05, 76M60, Conservation laws, Classical symmetry, Non-classical symmetry, Traveling wave

## Abstract

In this article, by using the Herman–Pole technique the conservation laws of the $(3+1)-$ Jimbo–Miwa equation are obtained, and then by using the Lie symmetry analysis all of the geometric vector fields of this equation are given. Also, the non-classical symmetries of the Jimbo–Miwa equation have been determined by applying nonclassical schemes. Eventually, the ansatz solutions of the Jimbo–Miwa equations utilizing the tanh technique have been offered.

## Introduction

Differential equations play a significant and key role in all sciences and disciplines; by using the analysis of these differential equations, the physical behaviors and manner of interaction and communication with the surrounding world can be discovered. So far, researchers have made great efforts to solve differential equations, including linear and nonlinear equations, and so on. One of the methods of achieving the solution is the conjoint analysis of the differential equations [14]. A study on Lie groups in the late nineteenth century was conducted by Sophus Lie [21], the Norwegian mathematician, inspired by the methodology of variate Galois, the French mathematician, an algebraist who had studies and articles on group theory, and used it for the analysis of polynomial equations. By using symmetries and applying them to the group’s work field, Lie was very interested in simplifying and eliminating the ambiguities of partial differential equations (PDEs) and was able to make a great revolution in science. Thus, Lie’s group analysis method is considered as one of the systematic methods employed to achieve the nonlinear differential equations’ solutions and plays an important role in this regard [26]. This method not only takes a big step toward obtaining differential equations by providing the appropriate tools, but also has approved its applicability by linking concepts such as conservation law and Lie’s symmetries in physics, mechanics, and other sciences [24, 25].

The conservation law in physics is the constancy of a physical quantity associated with a specific system during the evolution of it. Conservation laws of a differential equation have a very effective role in analyzing the properties of differential equations, more specifically finding exact solutions and expanding numerical methods to obtain more accurate answers and also finding nonlocal dependent devices [15, 16]. One of the most popular methods for obtaining conservation laws is Noether’s theorem method that expresses the relation between symmetries of a differential equation and its conservation laws by using Euler–Lagrange operator [13].

Although it is a very important result, there are limitations to the existence/nonexistence of any desired variations. Therefore, many efforts have been recently made to resolve this problem, and numerous other methods have been proposed to calculate the conservation law. Regardless of finding a variational problem, Ibragimov [15, 16] presented a method which is a generalization of the more fundamental theorem and obtained the conservation law formally based on the symmetries of the given system.

The conservation laws of differential equations can be obtained from different methods, including the classic method of Lie groups using Lie symmetries, in which one or more conservative laws can be constructed using asymmetry obtained from a system of differential equations. To achieve this, different techniques such as the direct technique, the Noether technique, the Boyer technique, the Ibragimov technique, the variational symmetries techniques, and the Herman–Pole technique can be used. The Hermann–Pole method provided in 2010 can largely remove the weaknesses of the direct method and calculate the density and flow of the conservation law in more systematic relations [13].

The JM equation was first introduced by Jimbo and Miwa [18] and then examined by a lot of scholars in various fields for obtaining its solutions [24, 25], integrability properties [11], symmetries [27], and so on. Scientists used the Jimbo–Miwa [18] equation to explain certain interesting $(3+1)$-dimensional waves in physics, and it is the second equation in the well-known Painlev’e hierarchy of integrable systems. Most recently, many researchers have investigated this equation. In the [28] Wazwaz obtained multiple-soliton solutions of the JM equation and its extended version.

Tang in [28] received its Pfaffian solution and extended Pfaffian solutions with applying the Hirota bilinear frame. Tang and Liang in [27] gained two kinds of variable separation solutions and plentiful nonlinear coherent structures with the aid of a multi-linear variable separation approach. By using the generalized Riccati equation mapping method, researchers presented rational solutions of $(3+1)$-JM equation [20]. In this paper, we utilize the conservation law and the symmetry method to get the solution of the $(3+1)$-dimensional partial differential equation in the following form:

1 $$ \mathrm{JM} : u_{x^{3}y}+3u_{x}u_{xy}+3u_{y}u_{x^{2}}+2u_{yt}-3u_{xz}=0, $$

which is a so-called *Jimbo–Miwa equation* in its potential frame. Bibi and co-workers [8] used the generalized Riccati equation mapping method for the space-time conformable Caudrey–Dodd–Gibbon equation by implementing the conformable derivative. Atangana and Araz [5] studied the spread of COVID-19 cases in Turkey and South Africa with an exhaustive statistical analysis. Alkahtani et al. [3] utilized the different operators of fractional differentiation with power law, exponential decay law, and Mittag-Leffler law to the Klein–Gordon equation with mass parameter and also investigated stability and the convergence of the used numerical scheme. Authors of [6] used the Crank–Nicholson scheme and investigated the stability and the convergence to the space fractional variable-order Schrödinger equation. Halder and co-authors [12] worked on Lie symmetry analysis and similarity solutions for the Jimbo–Miwa equation and generalizations. Also, Chauhan and co-authors [10] used the Lie group theoretic method to the similarity reduction and solitary wave solutions of (2 + 1)-dimensional Date–Jimbo–Kashiwara–Miwa equation. Khalique and Moleleki [19] studied a generalized first extended (3 + 1)- dimensional Jimbo–Miwa equation by using the $({G'}/{G})$-expansion method. In valuable works of scholars the conservation laws for a generalized Ito-type coupled KdV system [23], a new fourth-order integrable nonlinear equation [7], and a (3 + 1)-dimensional B-type Kadomtsev–Petviashvili equation [1], and fractional order coupled KdV system [17] were constructed by increasing the order of partial differential equations. In what follows, we show the Lie symmetry algebra for a JM equation by utilizing the Lie group analysis and find non-classical symmetries of the equation.

## Conservation laws for Jimbo–Miwa equation

There are various methods to obtain conservation laws. Herein, we employ the multiplier method. Taking the equation system

2 $$\begin{aligned} \mathbf{K}^{\sigma }[u]= \mathbf{K}^{\sigma } \bigl(x,u,\partial u,\ldots , \partial ^{k}u \bigr)=0, \quad \sigma =1, \ldots , N, \end{aligned}$$

via *n*-independent variables $x=(x^{1},\ldots ,x^{n})$ and *m*-dependent variables.

The conservation law for a system of PDEs (2) is a divergence expression that is defined as follows:

3 $$\begin{aligned} \mathbf{D}_{i}\aleph ^{i}[u]= \mathbf{D}_{1}\aleph ^{1}[u]+\cdots + \mathbf{D}_{n}\aleph ^{n}[u]=0. \end{aligned}$$

It is valid for all of the solutions of (3) that here $\aleph ^{i}[u]=\aleph ^{i}(x,u,\partial u,\ldots ,\partial ^{k}u)$ is named the fluxes of the conservation law and the order of conservation law is considered. The maximum degree of derivation in the flux expression. If $\Lambda _{\nu }[u]\mathbf{E}_{\nu }[u]\equiv \mathbf{D}_{i}\aleph ^{i}[u]$ for arbitrary functions *u*, then a set of multipliers coefficient function $\lbrace \Lambda _{\nu }[u]\rbrace _{\nu =1}^{l}=\lbrace \Lambda _{\nu }(x,u, \partial u,\ldots , \partial ^{k}u)\rbrace _{\nu =1}^{l}$ for the system $\mathbf{E}_{\nu }[u]=\mathbf{E}_{\nu }(x,u^{n})$, $\mathbf{E}(x,u)$ generates the divergence expression.

The Euler operator for $u^{J}$ is given by

$$\begin{aligned} E_{u^{j}}=\frac{\partial }{\partial u^{j}}-\mathbf{D}_{i} \frac{\partial }{\partial u^{j}_{i}}+\cdots +(-1)^{s} \mathbf{D}_{i_{1}} \cdots \mathbf{D}_{i_{s}} \frac{\partial }{\partial u^{j}_{i_{1}\cdots i_{s}}}+\cdots \end{aligned}$$

for $j=1,\ldots ,q $. That is, it makes zero the divergence expression $\mathbf{D}_{i}\aleph ^{i}[u] $. Relationships $\mathbf{E}_{u^{j}} \mathbf{F}(x,u,\partial u,\ldots ,\partial ^{s}u) \equiv 0$, $j=1,\ldots ,q $ hold for any free function $u(x)$ if and only if divergence equivalence

$$\begin{aligned} \mathbf{F} \bigl(x,u,\partial u,\ldots ,\partial ^{s}u \bigr)\equiv \mathbf{D}_{i} \aleph ^{i} \bigl(x,u,\partial u,\ldots , \partial ^{s-1}u \bigr) \end{aligned}$$

exists for $\aleph ^{i}(x,u,\partial u,\ldots ,\partial ^{s-1}u)$, $i=1,\ldots ,n $, functions.

### Theorem 2.1

([2])

*A set of local multipliers*$\Lambda _{\sigma }[u]_{\sigma =1}^{N}=\Lambda _{\sigma }(x,u, \partial u,\ldots ,\partial ^{l}u)_{\sigma =1}^{N}$*by arbitrary degree results in the conservation law for system* (2) *if and only if the following relationship for every arbitrary function* $u(x)$ *is maintained*:

4 $$\begin{aligned} \mathbf{E}_{u}^{j} \bigl[ \bigl(\Lambda _{\sigma } \bigl(x,u,\partial u,\ldots , \partial ^{l}u \bigr) \bigr)\mathbf{K}^{\sigma } \bigl(x,u,\partial u, \ldots ,\partial ^{k}u \bigr) \bigr] \equiv 0 ,\quad j=1,\ldots , m. \end{aligned}$$

*The equations obtained from* (2) *are a set of linear characteristic equations that can be solved by the set of all coefficients* $\Lambda _{\sigma }[u]_{\sigma =1}^{N} $.

Now, we can obtain the functional coefficients of the conservation law for the JM equation.

We can obtain the characteristic equation (3) for the JM equation from the following relation:

5 $$ E_{u} \bigl[\xi (x,y,z,t,u) (u_{x^{3}y}+3u_{x}u_{xy}+3u_{y}u_{x^{2}}+2u_{yt}-3u_{xz} ) \bigr]\equiv 0, $$

where $u(x,y,z,t)$ is an arbitrary function. By solving the characteristic equation obtained from (5), we write $\xi =a'(t)x +(k-3a(t))u_{x}+b(y,z)+c(z,t)$, where $a(t)$, $b(y,z)+c(z,t)$ are arbitrary coefficients. Then local multipliers are determined in the following frame:

$$\begin{aligned} \text{(i)}\quad a'(t)x-3a(t)u_{x},\qquad (\text{ii})\quad u_{x}, \qquad (\text{iii})\quad b(y,z), \qquad (\text{iv})\quad c(z,t) . \end{aligned}$$

Each of the functional coefficients $\Lambda _{i}=\xi _{i}$ is a conservation law $D_{x}\Psi +D_{y}\Phi +D_{z}\Omega +D_{t}\theta =0$ with a determining form

$$\begin{aligned} D_{x}\Psi +D_{y}\Phi +D_{z}\Omega +D_{t}\theta =\xi . (u_{x^{3}y}+3u_{x}u_{xy}+3u_{y}u_{x^{2}}+2u_{yt}-3u_{xz} ). \end{aligned}$$

We use the four-dimensional homotopy operator $(\mathcal{H}_{u}^{x}f,\mathcal{H}_{u}^{y}f,\mathcal{H}_{u}^{z}f, \mathcal{H}_{u}^{t}f)$ to calculate the Ψ, Φ, Ω, and *θ*, where x-component is defined as follows:

6 $$ \mathcal{H}_{u}^{x}f= \int _{0}^{1}\frac{1}{\lambda } \Biggl(\sum _{j=1}^{q} \mathcal{I}_{u^{j}}^{x}f \Biggr)[\lambda u]\,d\lambda . $$

The *t*, *y*, and *z*- components can also be defined similar to (6). In (6), $\mathcal{I}_{u^{j}}^{x}f$ is given by

7 $$\begin{aligned} \sum_{E} \biggl(\sum _{F} B^{(x)} u^{j}_{x^{i_{1}}y^{i_{2}}z^{i_{3}}t^{i_{4}}} {\mathbf{D}}_{k_{1},k_{2},k_{3},k_{4}}^{i_{1},i_{2},i_{3},i_{4}} \biggr) \frac{\partial f}{\partial u^{j}_{x^{k_{1}}y^{k_{2}}z^{k_{3}}t^{k_{4}}}} , \end{aligned}$$

where

$$\begin{aligned}& E:= \bigl\{ 1\leq k_{1}\leq M_{1}^{j}, 0\leq k_{2}\leq M_{2}^{j}, 0 \leq k_{3}\leq M_{3}^{j}, 0\leq k_{4}\leq M_{4}^{j} \bigr\} , \\& F:=\{ 0\leq i_{1}\leq k_{1}-1, 0\leq i_{2} \leq k_{2}, 0\leq i_{3} \leq k_{3}, 0\leq i_{4}\leq k_{4}\}, \\& {\mathbf{D}}_{k_{1},k_{2},k_{3},k_{4}}^{i_{1},i_{2},i_{3},i_{4}}:=(-D_{x})^{k_{1}-i_{1}-1}(-D_{y})^{k_{2}-i_{2}}(-D_{z})^{k_{3}-i_{3}}(-D_{t})^{k_{4}-i_{4}}, \end{aligned}$$

and $M^{j}_{1}$, $M^{j}_{2}$, $M^{j}_{3}$, $M^{j}_{4}$ are the order of $u^{j}$ with respect to *x*, *y*, *z*, and *t* in *f*, which in the (JM) equation $j=1$, $M^{j}_{1}=3$, $M^{j}_{2}=1$, $M^{j}_{3}=1$, $M^{j}_{4}=1$. Also, the combinatorial coefficient is evaluated by

$$\begin{aligned} B^{(x)} = & B(i_{1},i_{2},i_{3},i_{4},k_{2},k_{3},k_{3}) \\ = & C(i_{1}+i_{2}+i_{3}+i_{4},i_{1}) C(i_{2}+i_{3}+i_{4},i_{2}) C(i_{3}+i_{4},i_{3}) \\ &{}\times C(k_{1}+k_{2}+k_{3}+k_{4}-i_{1}-i_{2}-i_{3}-i_{4}-1,k_{1}-i_{1}-1) \\ &{}\times C(k_{2}+k_{3}+k_{4}-i_{2}-i_{3}-i_{4},k_{2}-i_{2}) C(k_{3}+k_{4}-i_{3}-i_{4},k_{3}-i_{3}) \\ &{} \div C(k_{1}+k_{2}+k_{3}+k_{4},k_{1}) C(k_{2}+k_{3}+k_{4},k_{2}) C(k_{3}+k_{4},k_{3}). \end{aligned}$$

Consider $\mathcal{I}_{u^{j}}^{y}f$, $\mathcal{I}_{u^{j}}^{z}f$, and $\mathcal{I}_{u^{j}}^{t}f$ same as $\mathcal{I}_{u^{j}}^{x}f$. Now, we determine conserved quantities *ψ*, *ϕ*, Ω, and Θ which conclude of multiplier $\varepsilon =a'(t)x-3a(t)u_{x}$. So we consider

$$\begin{aligned} f= \bigl(a'(t)x-3a(t)u_{x} \bigr) (u_{x^{3}y}+3u_{x}u_{xy}+3u_{y}u_{x^{2}}+2u_{yt}-3u_{xz} ). \end{aligned}$$

By using (6) and (7), we have

8 $$\begin{aligned} \mathcal{I}_{u^{j}}^{x}f =& \frac{3a}{4} \bigl(6uu_{xz}-u_{y}u_{x^{3}}-uu_{x^{2}y}+3u_{x^{2}}u_{xy}-5u_{x}u_{x^{2}y} \\ &{} -18u_{y}u_{x}^{2}+6u_{z}u_{x}-8uu_{yt} \bigr)-a_{t} \biggl(3uu_{y}+ \frac{1}{2}u_{xy} \biggr) \\ &{} + \frac{3xa_{t}}{2} \biggl(\frac{1}{2}u_{x^{2}y}-u_{z}+3u_{x}u_{y}-uu_{xy} \biggr), \\ \mathcal{I}_{u^{j}}^{y}f =&\frac{3xa_{t}}{2} \biggl(uu_{x^{2}}+u_{x}^{2}+ \frac{1}{6}u_{x^{3}} \biggr)+\frac{3a}{2} \bigl(2uu_{xt}-2u_{x}u_{t}-3u_{x}^{3}-u_{x}u_{x^{3}} \bigr) \\ &{} +\frac{3a}{4} \bigl(uu_{x^{4}}+u_{x^{2}}^{2} \bigr)+\frac{1}{4}(3uu_{x}-u_{x^{2}}+4xu_{t})-xua_{tt}, \\ \mathcal{I}_{u^{j}}^{z}f =&\frac{3a_{t}}{2}(u-xu_{x})- \frac{3a}{2} \bigl(uu_{x^{2}}+u_{x}^{2} \bigr), \\ \mathcal{I}_{u^{j}}^{t}f =&3a(uu_{xy}-u_{x}u_{y})+a_{t}u_{y}x. \end{aligned}$$

Substituting (8) into (6), we have

$$\begin{aligned} \Psi :=&\mathcal{H}_{u^{j}}^{x}f = \frac{3a}{8} \bigl(6uu_{xz}-u_{y}u_{x^{3}}-uu_{x^{2}y}+3u_{x^{2}}u_{xy}-5u_{x}u_{x^{2}y}-6u_{y}u_{x}^{2} \\ &{} + 6u_{z}u_{x}-8uu_{yt} \bigr)+ \frac{3xa_{t}}{4} (u_{x^{2}y}-2uz+3u_{x}u_{y}-uu_{xy} )-\frac{1}{2}(3uu_{y}+u_{xy}), \\ \Phi :=&\mathcal{H}_{u^{j}}^{y}f = \frac{xa_{t}}{4} \bigl(3uu_{x^{2}}+3u_{x}^{2}+u_{x^{3}} \bigr)+ \frac{a_{t}}{4}(3uu_{x}-u_{x^{2}}+4xu_{t}) \\ &{} +\frac{3a}{8} \bigl(4uu_{xt}-4u_{x}u_{t}-4u_{x}^{3}+uu_{x^{4}}-2u_{x}u_{x^{3}}+u_{x^{2}}^{2} \bigr)-xua_{tt}, \\ \Omega :=&\mathcal{H}_{u^{j}}^{z}f=\frac{3a_{t}}{2}(u-xu_{x})- \frac{9a}{4} \bigl(uu_{x^{2}}+u_{x}^{2} \bigr), \\ \Theta :=&\mathcal{H}_{u^{j}}^{t}f =\frac{3a}{2}(uu_{xy}-u_{x}u_{y})+a_{t}u_{y}x. \end{aligned}$$

So, the conservation law of the JM equation for the case $\varepsilon =a'(t)x-3a(t)u_{x}$ is $D_{x}\Psi + D_{y}\Phi +D_{z}\Omega +D_{t}\Theta =0$. Now, by using a similar method for other cases, we can find all the conservation laws. Therefore, we obtain in the case $\xi =u_{x}$:

$$\begin{aligned}& D_{x} \bigl(u_{y} \bigl(12u_{x}^{2}+u_{x^{3}} \bigr)+u(2u_{yt}-6u_{xz}+u_{x^{3}y})+2u_{x}(5u_{x^{2}y}-3u_{z})-3u_{xy}u_{x^{2}} \bigr) \\& \quad {}+D_{y} \bigl(4u_{x}(2u_{t}+u_{x^{3}})-2u(2u_{xt}+u_{x^{3}})+4u_{x}^{3}-u_{x^{2}}^{2} \bigr) \\& \quad {}+6D_{z} \bigl(uu_{x^{2}}-u_{x}^{2} \bigr)+4D_{t} (u_{x}u_{y}-uu_{xy} )=0, \end{aligned}$$

and in the case $\xi =b(y,z)$:

$$\begin{aligned}& 4D_{x} \bigl(3b(uu_{xy}+3u_{x}u_{y}-2u_{z}+u_{x^{2}y})+b_{y}(u_{x^{2}}+uu_{x})-6b_{z}u \bigr) \\& \quad {}-D_{y} \bigl(3b \bigl(4uu_{x^{2}}+3u_{t}+4u_{x}^{2}+12u_{x^{3}} \bigr) \bigr)+24D_{z} (bu_{x} )+16D_{t} (b_{y}u-bu_{y} )=0, \end{aligned}$$

and finally for $\xi =c(z,t)$:

$$\begin{aligned}& 3 D_{x} \bigl(c(uu_{xy}+3u_{x}u_{y}-2u_{z}+u_{x^{2}y})+2c_{z}u \bigr) \\& \quad {}-D_{y} \bigl(c \bigl(3uu_{x^{2}}+4u_{t}+3u_{x}^{2}+9u_{x^{3}} \bigr)-4c_{t}u \bigr)+6D_{z} (cu_{x} )-4D_{t} (cu_{y} )=0. \end{aligned}$$

## Classical symmetries of Jimbo–Miwa equation

The symmetry group of equation (1) is made of the vector field of the following form:

9 $$ X=\xi _{x}\partial _{x}+\xi _{y} \partial _{y}+\xi _{z} \partial _{z}+ \xi _{t} \partial _{t}+\eta _{u} \partial _{u}. $$

The fourth order prolongation *X* is determined by the following vector field:

10 $$\begin{aligned} X^{(4)} =&X+\varphi ^{x} \partial _{u_{x}}+\varphi ^{y}\partial _{u_{y}}+ \varphi ^{z}\partial _{u_{z}}+\varphi ^{t} \partial _{u_{t}} \\ &{} +\varphi ^{x^{2}}\partial _{u_{x^{2}}}+ \varphi ^{xt} \partial _{u_{xt}}+\cdots +\varphi ^{tttt} \partial _{u_{tttt}}, \end{aligned}$$

where $\varphi ^{\iota }=D_{\iota }Q+\xi u_{x\iota }+\eta u_{t\iota }$, $\varphi ^{\iota \jmath }=D_{\imath }(D_{\jmath }Q)+\xi u_{x\imath \jmath }+\eta u_{t\imath \jmath }$, $Q=\varphi -\xi u_{x}-\eta u_{t}$ is the characteristic of *X* given by (10) and $D_{i}$ indicates total derivative. Thus, equation (1) admits a Lie point symmetry *X* if $X^{(4)}[u_{x^{3}y}+3u_{x}u_{xy}+3u_{y}u_{x^{2}}+2u_{yt}-3u_{xz}]=0$, where $X^{(4)}$ denotes the fourth prolongation of *X*. We can find the determining equations for the symmetry group of the general JM equation as follows:

$$\begin{aligned}& \xi ^{1}_{y} ,\xi ^{3}_{y},\xi ^{4}_{y},\xi ^{4}_{z},\xi ^{2}_{t}, \xi ^{3}_{t},\xi ^{2}_{x},\xi ^{3}_{x},\xi ^{4}_{x},\xi ^{1}_{u},\xi ^{2}_{u}, \xi ^{3}_{u},\xi ^{4}_{u}, \xi ^{1}_{t^{2}z},\xi ^{3}_{z^{2}},\eta ^{1}_{zu}, \eta ^{1}_{tu},\eta ^{1}_{u^{2}}=0, \\& 3\xi ^{2}_{z^{2}}=4\xi ^{1}_{tz}, \qquad \xi ^{1}_{x} =- \eta ^{1}_{u}, \qquad \xi ^{1}_{z} =- \eta ^{1}_{y}, \qquad \xi ^{4}_{t} =-3 \eta ^{1}_{u}, \\& 2\xi ^{1}_{t} - 3\xi ^{2}_{z}=3 \eta ^{1}_{x}, \qquad \xi ^{2}_{y} - \xi ^{3}_{z}=2 \eta ^{1}_{u}. \end{aligned}$$

By solving this system of PDEs, we find that

$$\begin{aligned}& \xi _{1}=c_{1}x +3tF_{1}'v+ F_{2}t+F_{3}z, \qquad \xi _{2} =-4F_{1}z+ (3c_{3} t -2 c_{1})y ,\qquad \xi _{3}= 3c_{3}z+ c_{4} , \\& \xi _{4}= 3c_{1}t+ c_{2} ,\qquad \eta _{1} = 3ytF_{1}''z- yF_{3}'z+ 4xF_{2}'t- 12xF_{1}'z -2c_{2} + F_{4}(z,t), \end{aligned}$$

where $c_{i}$, $i=1, \ldots , 4$, are arbitrary constants and $F_{i}(t)$, $i=1, \ldots , 4$, are arbitrary smooth functions. Solving the above determining equations, we reach the following Lie point symmetry generators:

$$\begin{aligned}& X_{1}= x\partial _{x}-2y\partial _{y}+3t \partial _{t}-u\partial _{u}, \qquad X_{5}^{f}=3tf_{t} \partial _{x}+4f\partial _{y}-(2xf_{t}+3ytf_{tt}) \partial _{u}, \\& X_{2}=\partial _{t}, \qquad X_{6}^{g}=3g \partial _{x}+2xg_{t} \partial _{u}, \\& X_{3}= y\partial _{y}+z\partial _{z}, \qquad X_{7}^{h}=h\partial _{x}-yh_{z} \partial _{u}, \\& X_{4}=\partial _{z}, \qquad X_{8}^{d}= d\partial _{u}, \end{aligned}$$

where $f=f(z)$, $g=g(t)$, $h=h(z)$, and $d=d(z,t)$ are arbitrary smooth functions. Having functional coefficients, these vector fields produce a Lie pseudo-group $\mathbf{g}=L$(G). This Lie pseudo-algebra **g** has a 4− sub-algebra $\mathbf{L}_{4} \simeq \mathbf{af}(1)\times \mathbf{af}$(1) made by $X_{1}, \ldots , X_{4}$ and an infinite dimensional ideal $\mathbf{L}_{\infty }$ generated by $X_{5}^{f}$, $X_{6}^{g}$, $X_{7}^{h}$, $X_{8}^{d}$. Therefore $\mathbf{L} \simeq \mathbf{L}_{4}\rtimes \mathbf{L}_{\infty }$. The commutation relations satisfied by generators above are shown Table 1.

**Table 1:** The commutator table of ${\mathfrak{g}}$

[⋅,⋅]	$X_{1}$	$X_{2}$	$X_{3}$	$X_{4}$	$X_{5}^{f}$	$X_{6}^{g}$	$X_{7}^{h}$	$X_{8}^{d}$
$X_{1}$	0	$-3X_{2}$	0	0	$2X_{5}^{f}$	$X_{6}^{3t g'-g}$	$-X_{7}^{h}$	$X_{8}^{3t d_{t}+d}$
$X_{2}$	$3X_{2}$	0	0	0	$X_{7}^{3f'/4}$	$X_{6}^{g'}$	0	$X_{8}^{d_{t}}$
$X_{3}$	0	0	0	$-X_{4}$	$X_{5}^{zf'-f}$	0	$X_{7}^{zh'}$	$X_{8}^{zd_{z}}$
$X_{4}$	0	0	$X_{4}$	0	$X_{5}^{f'}$	0	$X_{7}^{h'}$	$X_{8}^{d_{z}}$
$X_{5}^{\tilde{f}}$	$-2X_{5}^{\tilde{f}}$	$-X_{7}^{3\tilde{f}'/4}$	$-X_{5}^{z\tilde{f}'-\tilde{f}}$	$-X_{5}^{\tilde{f}'}$	$X_{5}^{3t(f\tilde{f}''-\tilde{f}f'')/4}$	$X_{8}$	$X_{8}$	0
$X_{6}^{\tilde{g}}$	$-X_{6}^{3t \tilde{g}'-\tilde{g}}$	$-X_{6}^{\tilde{g}'}$	0	0	$-X_{8}$	$X_{8}$	$X_{8}$	0
$X_{7}^{\tilde{h}}$	$X_{7}^{\tilde{h}}$	0	$-X_{7}^{z\tilde{h}'}$	$-X_{7}^{\tilde{h}'}$	$-X_{8}$	$-X_{8}$	0	0
$X_{8}^{\tilde{d}}$	$-X_{8}^{3t \tilde{d}_{t}+\tilde{d}}$	$-X_{8}^{\tilde{d}_{t}}$	$-X_{8}^{z\tilde{d}_{z}}$	$-X_{8}^{\tilde{d}_{z}}$	0	0	0	0

Consider $\mathbf{Ad}(\exp (s. X).Y)=\sum_{k=0}^{\infty }(-s)^{k}(k!)^{-1} \mathbf{ad}^{k}_{X}Y$, where *s* is the group parameter. An adjoint action is considered for Lie algebra **g**, so we consider the following theorem.

### Theorem 3.1

*The optimal system of* 1-*subalgebras for JM is*

$$\begin{aligned}& 1)\quad \langle X_{3} \rangle , \qquad 2) \quad \langle X_{4}\rangle , \qquad 3)\quad \langle X_{1}+ X_{4} \rangle , \qquad 4) \quad \langle X_{1}- X_{4} \rangle , \qquad 5)\quad \langle X_{2}+ X_{3} \rangle , \\& 6)\quad \langle X_{2}- X_{3} \rangle , \qquad 7)\quad \langle X_{2}+ X_{4} \rangle , \qquad 8)\quad \langle X_{2}- X_{4} \rangle , \qquad 9)\quad \langle a_{1} X_{1}+ a_{3} X_{3} \rangle . \end{aligned}$$

### Proof

Let $\mathfrak{L}_{4}$ be the symmetry algebra of Eq. (1) by applying adjoint and $X= a_{1} X_{1}+ \cdots + a_{4} X_{4}$, that is, it is a nonzero vector field of $\mathfrak{L}_{4}$. We can simplify as many of the coefficients $a_{1},\ldots ,a_{4}$ as possible through proper adjoint applications on *X*. So we have the following:

- If we assume that $a_{2}\neq 0 $, by scaling $X_{2}$ and then assuming $a_{4}=0 $ and scaling $X_{1} $, *X* is reduced to $X_{3} $, $X_{2}+ X_{3} $, and $X_{2}- X_{3} $. In this case if $a_{4}\neq 0 $ and by scaling $X_{3} $, *X* is reduced to $X_{4} $, $X_{2}+ X_{4} $ and $X_{2}- X_{4} $.
- If we assume that $a_{2}= 0 $ and $a_{4}\neq 0 $, by scaling $X_{4} $ and $X_{3} $, *X* is reduced to $X_{4} $, $X_{1}+ X_{4} $, and $X_{1}- X_{4} $. If we assume that $a_{2}=a_{4}= 0 $, *X* is reduced to $a_{1} X_{1}+ a_{3} X_{3} $.

 □

Similarly, we can classify 2-sub-algebra and 3-sub-algebra. For convenience, we can use the normal sub-algebra to make the sub-algebra.

## Group invariant solutions and similarity reduction of JM

Equation (1) can be considered as a sub-manifold of the jet space $J^{4}(\mathbb{R}^{4},\mathbb{R}^{3})$. Thus, according to [26], we can obtain the most general group invariant solutions of (1). Since group transformations are generated by infinitesimal generators $X_{i}$, we need to solve the following system of differential equations:

$$\begin{aligned} g_{1}^{s}&= \bigl(xe^{s}, ye^{-2s}, z, te^{3}s, u \bigr),\qquad g_{2}^{s}= (x, y, z, t+s, u ), \\ g_{3}^{s}&= \bigl(x, ye^{s}, ze^{s}, t, u \bigr), \qquad g_{4}^{s}= (x, y, z+s, t, u ), \\ g_{5}^{s}&= \biggl(x+\frac{3st}{4}f_{z}, y+sf, z, t, u-\frac{3st}{8}(2y+sf)f_{zz}- \frac{3}{s^{2} t}f_{z}^{2}- \frac{sx}{2}f_{z} \biggr), \\ g_{6}^{s}&= \biggl(x+sg_{t}, y, z, t, u+ \frac{1}{3}g_{t} \bigl(s^{2}g+2sx \bigr) \biggr), \\ g_{7}^{s}&= (x+sh, y, z, t, u-syh_{z} ), \qquad g_{8}^{s}= (x, y, z, t, u+sd ), \end{aligned}$$

where the transformed point is $g_{1}^{s}(x, y, z, t):=\exp (sX_{i})(x, y, z, t)$. So, corresponding to the above invariant transformations, the group invariant solutions result is expressed in the following theorem.

### Theorem 4.1

*If*$u(t, x, y, z)$*is a solution of the JM equation*, *then the following functions are solutions of the JM equation as well*:

$$\begin{aligned} &\varphi _{1}=e^{-s}f \bigl(x e^{-s}, y e^{2s}, z ,te^{-3s} \bigr), \qquad \varphi _{2}=f(x ,y ,z ,t-s ), \\ &\varphi _{3}=f \bigl(x, y e^{-s}, z e^{-s}, t \bigr), \qquad \varphi _{4}=f(x ,y ,z-s ,t ), \\ &\varphi _{5}=f \biggl(x-\frac{3st}{4}f_{z}, y-sf, z, t \biggr)+s^{2}tff_{zz}+ \frac{s^{2}t}{2}f_{z}^{2}-2 syt f_{zz}-\frac{3}{4xs} f_{z}, \\ &\varphi _{6}=f(x-sg,y ,z ,t )-\frac{s^{2}}{3}gg_{t}+ \frac{2xs}{3} g_{t},\qquad \varphi _{7}=f(x-sh ,y ,z ,t )-syh_{z}, \\ &\varphi _{8}=f(x ,y ,z ,t )+sd. \end{aligned}$$

As the JM equation is expressed in the coordinates $(x,y,z,t,u)$, we need to look for specific coordinates to reduce the equation. These coordinates, which are represented by $(r,q,w,f)$, are obtained by finding independent invariants which correspond to the infinitesimal symmetry generators. Finally, we obtain the reduced equation by using the new coordinates and applying the chain rule. We now obtain some invariant group solutions for the JM equation. We must solve the PDEs $X[I]=0$ to determine independent invariants *X*, so we have

$$\begin{aligned} (X_{2}+X_{3}+X_{4})I = yI_{y}+(z+1)I_{z}+I_{t}=0. \end{aligned}$$

We must solve the associated characteristic ODE $dt=dy/y=dx/0=dz/(z+1)=du/0$ for solving the above PDE. Hence, we obtain four functionally independent invariants $r=(z+1)/y$, $q=-\ln (y)+t$, $w=x$ and $f=u$, where *v* is a function of *r*, *q*, and *w*. By using the chain rule and the fact that $u=f(r,q,w)$ and then appending them into the JM equation, we obtain the following reduced equation:

11 $$\begin{aligned} r{\mathcal{R}}(f_{w^{3}})+3f_{w}{ \mathcal{R}}(f_{w})+3f_{w^{2}}{\mathcal{R}}(f)+2{ \mathcal{R}}(f_{q})+3f_{r^{2}}=0, \end{aligned}$$

where ${\mathcal{R}}:=r\partial _{r}+\partial _{q}$. The polynomial solution of this equation (11) is $f(w)= c_{1}w^{2}+c_{2}w+c_{3}$.

By a similar argument, for $X_{1}+X_{2}+X_{3}$, we can obtain the reduced form of the equation as follows:

$$\begin{aligned} 8w^{3}f^{3}(wf+9) - w \bigl(8w^{2}+(f-25)w-1 \bigr) f^{2} =48w^{2} f^{\prime \,2}+(5wf-20w+3) f', \end{aligned}$$

and other polynomial solutions as $f(r,w)= c_{1}rw^{2}+c_{2}w$ and

$$\begin{aligned} f(r,q)= c_{1}q^{4}+(c_{2}r+c_{3})q^{3}+(c_{4}r+c_{5})q^{2}+(c_{6}r+c_{7})q+c_{8}r+c_{9}, \end{aligned}$$

where $c_{1},\ldots ,c_{9}$ are arbitrary parameters.

Besides, we could use 3-sub-algebras for reducing the equation. We use 1-sub-algebra to obtain 3-sub-algebras because 3-sub-algebras are vertical to 1-sub-algebras. For instance, with the selection of several algebras of 3-sub-algebras, we reduce the equation. By choosing $\langle X_{4}, X_{2}, X_{1} \rangle $, the equation is reduced to

$$\begin{aligned} 8k^{2}f^{3}(kf+3)+6k \bigl(8k f'-f+1 \bigr)f''-3ff'=0, \end{aligned}$$

where $k=yx^{2} $, and also selecting $\langle X_{1}, X_{3}, X_{4}\rangle $ leads to $f=\varphi (t/x^{3}) $ and the equation becomes zero. By selecting $\langle X_{1}, X_{2}, X_{3}\rangle $, the equation is reduced to the following:

$$\begin{aligned} 8f^{3}k^{3}(kf+9)=6k \bigl(4k^{2}f'+kf-25k-1 \bigr)f''+48 k^{2}ff^{\prime \,2} +3(5kf-20k-3)f'. \end{aligned}$$

## Non-classical symmetries of JM

Non-classic symmetry is another way to determine some other solutions for a PDE and ODE system. Here, we use the conventional method to obtain the non-classical symmetry of the JM equation according to the compatibility of the evolutionary equations [4, 9].

In a non-classical way, first, we add the conditions of the invariance surface to the equation and then apply the classical symmetry method. So, we express $X^{(4)}\Delta _{1}\equiv 0\mod \Delta _{1}=0$, $\Delta _{2}=0$, where *X* is defined in (9) and $\Delta _{1}$ and $\Delta _{2}$ are given as $\Delta _{1}:=u_{x^{3}y}+3u_{x}u_{xy}+3u_{y}u_{x^{2}}+2u_{yt}-3u_{xz}$ and $\Delta _{2}:=\eta u_{t}+\xi u_{x}+\beta u_{y}+\zeta u_{z}-\varphi $. By using non-classical methods on the JM, we consider the following equations:

$$\begin{aligned} & \eta _{x},\eta _{y},\eta _{t},\eta _{u},\zeta _{x},\zeta _{y}, \zeta _{z},\zeta _{t},\zeta _{u},\xi _{x},\xi _{y},\xi _{u},\xi _{t^{2}z}, \phi _{u}=0, \\ & \eta _{z}^{2}=4\xi _{tz}, \qquad \phi _{x}=2\xi _{t}-\eta _{z}, \qquad \phi _{y}=-3\xi _{z}. \end{aligned}$$

Therefore we have these solutions:

$$\begin{aligned} \xi &=h+3tg_{z}+3f, \qquad \eta =4g+3c_{1}z+c_{2}, \\ \zeta &=3c_{1}, \qquad \phi =2(f_{t}-g_{z}-c_{1})-h_{z}+3tyg_{zz}+d, \end{aligned}$$

where $c_{1}$ and $c_{2}$ are free amounts and $f = f(t)$, $g = g(z)$, $h = h(z)$, and $d = d(z,t)$ are arbitrary smooth functions.

## Some ansatz exact solutions of JM

Herein, we perform one of the most important ansatz methods (the tanh-function method [22]) to gain exact traveling wave solutions of this nonlinear system of PDEs. For this, a new variable $\tau =\tanh (c_{1}x+c_{2}y+c_{3}z+c_{4}t+c_{5})$, where $c_{i}$ are arbitrary constants, is considered. By placing this expression in equation (1), we get

$$\begin{aligned}& c_{1}^{2}c_{2} \bigl(\tau ^{2}-1 \bigr)^{2} \bigl(c_{1} \bigl(\tau ^{2}-1 \bigr)u_{\tau ^{4}}+12 \tau c_{1}u_{\tau ^{3}}-6u_{\tau }u_{\tau \tau } \bigr) \\& \quad {} + \bigl(\tau ^{2}-1 \bigr) \bigl( 4c_{1}^{3}c_{2} \bigl(9\tau ^{2}-2 \bigr)-3c_{1}c_{3}+2c_{2}c_{4} \bigr) u_{\tau \tau } \\& \quad {} -12\tau c_{1}^{2}c_{2} \bigl(\tau ^{2}-1 \bigr) u_{\tau }^{2}+2\tau \bigl( 4c_{1}^{3}c_{2} \bigl(3 \tau ^{2}-2 \bigr)-3c_{1}c_{3}+2c_{2}c_{4} \bigr) u_{\tau }=0. \end{aligned}$$

Then, using the ansatz $u=A_{0}+A_{1}\tau +A_{2}\tau ^{2}+A_{3}\tau ^{3}$, where $A_{i}$ are arbitrary constants, we obtain the exact solution by using required simplifications and linear algebra:

12 $$\begin{aligned} u=2c_{1}\tanh \biggl( c_{4}t+c_{1}x+c_{2}y+{ \frac{2c_{2}(2c_{1}^{2}+c_{4})}{3c_{1}}}z+c_{5} \biggr)+c_{0}. \end{aligned}$$

**Figure 1:**
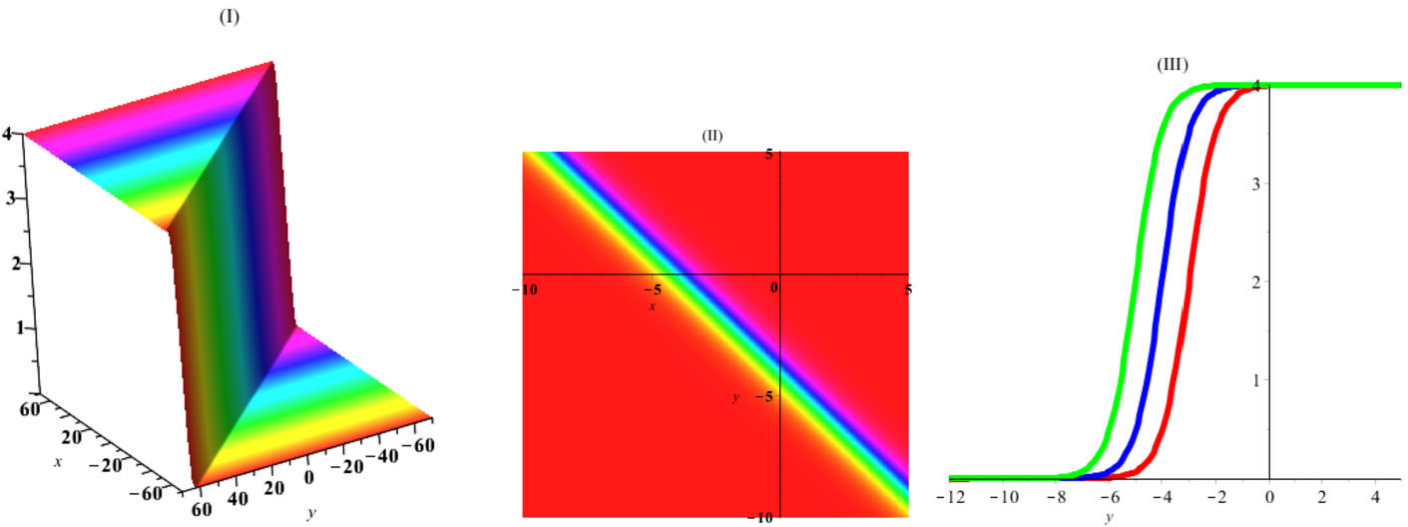
The plot of solitary (12) with amounts $c_{0} = 2$, $c_{1}= 1$, $c_{2}= 1$, $c_{3}= 1$, $c_{4}= 1$, $c_{5}= 1$, $t = 1$, $z = 1$

**Figure 2:**
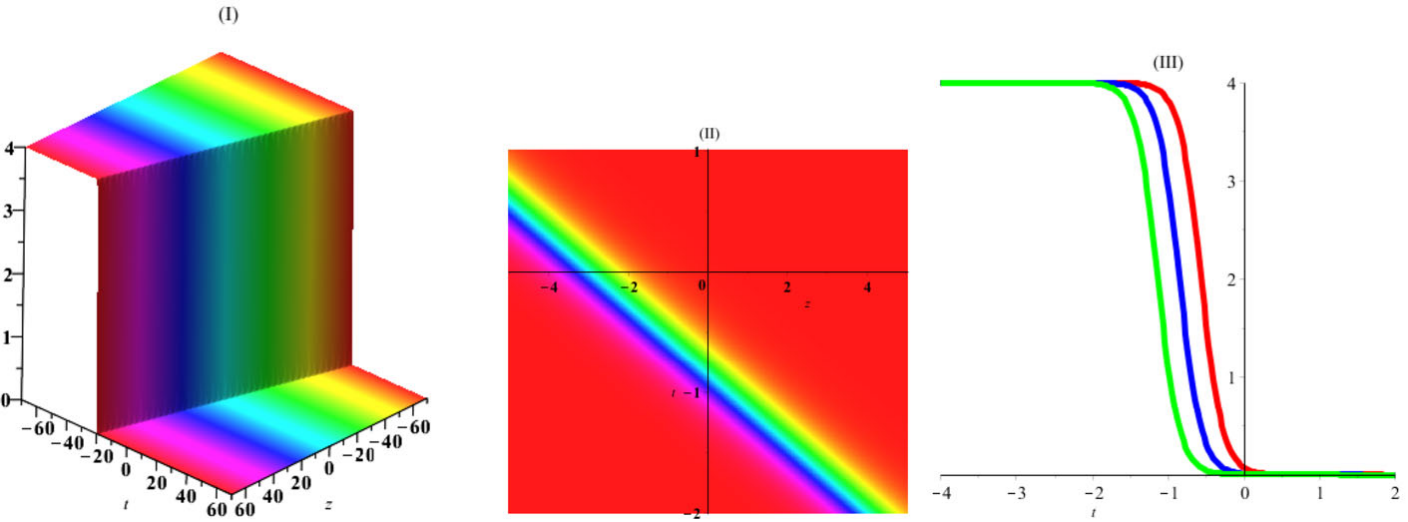
The plot of solitary (12) with amounts $c_{0} = 2$, $c_{1}= 1$, $c_{2}= 1$, $c_{3}= 1$, $c_{4}= 1$, $c_{5}= 1$, $x = 1$, $y = 1$

**Figure 3:**
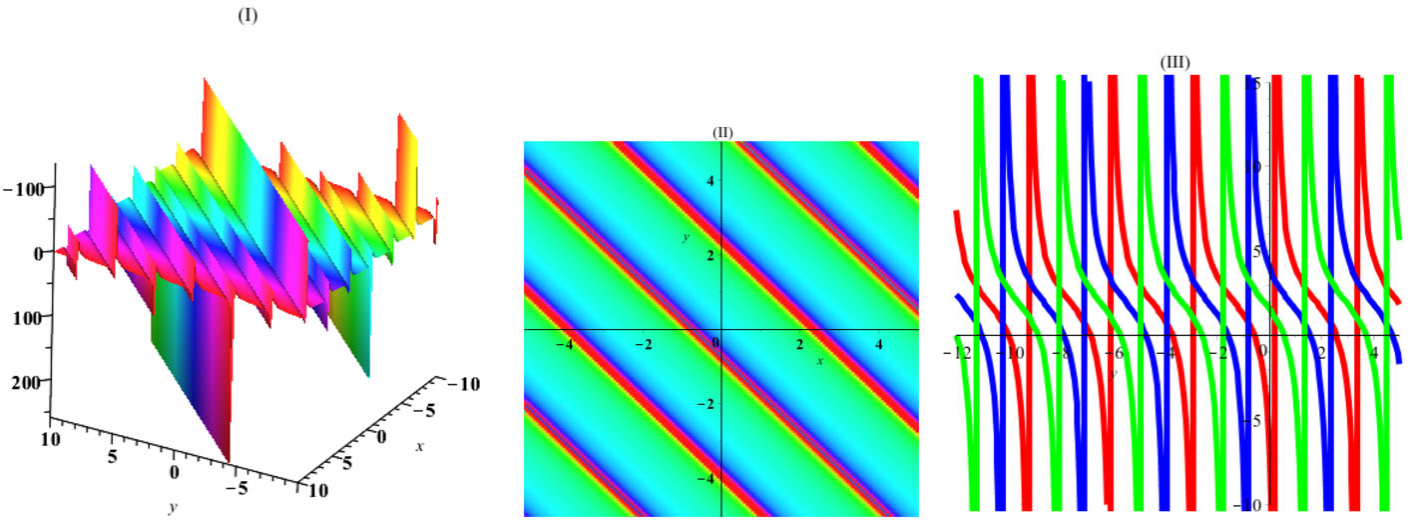
The plot of solitary (13) with amounts $c_{0} = 2$, $c_{1}= 1$, $c_{2}= 1$, $c_{3}= 1$, $c_{4}= 1$, $c_{5}= 1$, $t = 1$, $z = 1$

**Figure 4:**
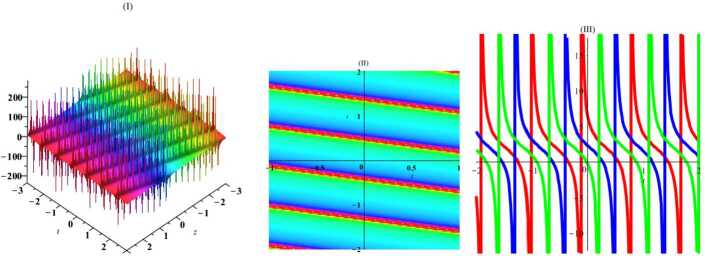
The plot of solitary (13) with amounts $c_{0} = 2$, $c_{1}= 1$, $c_{2}= 1$, $c_{3}= 1$, $c_{4}= 1$, $c_{5}= 1$, $x = 1$, $y = 1$

In Figs. 1–4, the solutions have been plotted for some constant coefficients. By choosing suitable amounts, the dynamical structures of hyperbolic wave solutions are presented in Figs. 1 and 2 including three-dimensional, density, and *y*-curves plot in Fig. 1 and *t*-curves plot in Fig. 2. Moreover, by choosing suitable amounts, the dynamical structures of periodic wave solutions are presented in Figs. 3 and 4 including three-dimensional, density, and *y*-curves plot in Fig. 3 and *t*-curves plot in Fig. 4.

Similarly, ansatz $\tau =\tan (c_{1}x+c_{2}y+c_{3}z+c_{4}t+c_{5})$, where $c_{i}$ are arbitrary constants, leads to

13 $$\begin{aligned} u= -2c_{1}\tan \biggl(c_{1}x+c_{2}y+c_{3}z+ \frac{c_{1}(4c_{1}^{2}c_{2}+3c_{3})}{2c_{2}}t+c_{5} \biggr)+c_{0}. \end{aligned}$$

## Conclusions

In this article, we obtained the conservation laws for JM equation that give unequivocal expressions of conserved quantities. Then, we attempted to show the Lie symmetry algebra for JM equation by utilizing the Lie group analysis. Further, we found non-classical symmetries of the equation. Finally, we obtained the exact solitary wave solution of JM equation by utilizing the tanh-function technique. The graphes were plotted containing 3D plot, density plot, and 2D plot. The results are beneficial to the study of the mathematics physics, fluid dynamics, and applied mechanics. All calculations in this paper have been made quickly with the aid of the Maple.

## Data Availability

The data sets supporting the conclusions of this article are included in the article.
